# TMSCNet: A three-stage multi-branch self-correcting trait estimation network for RGB and depth images of lettuce

**DOI:** 10.3389/fpls.2022.982562

**Published:** 2022-08-31

**Authors:** Qinjian Zhang, Xiangyan Zhang, Yalin Wu, Xingshuai Li

**Affiliations:** ^1^School of Mechanical Electrical Engineering, Beijing Information Science and Technology University, Beijing, China; ^2^Lushan Botanical Garden, Chinese Academy of Sciences, Jiujiang, China

**Keywords:** NMSE, master model, auxiliary model, automatic correction, pseudo color map, high-throughput

## Abstract

Growth traits, such as fresh weight, diameter, and leaf area, are pivotal indicators of growth status and the basis for the quality evaluation of lettuce. The time-consuming, laborious and inefficient method of manually measuring the traits of lettuce is still the mainstream. In this study, a three-stage multi-branch self-correcting trait estimation network (TMSCNet) for RGB and depth images of lettuce was proposed. The TMSCNet consisted of five models, of which two master models were used to preliminarily estimate the fresh weight (FW), dry weight (DW), height (H), diameter (D), and leaf area (LA) of lettuce, and three auxiliary models realized the automatic correction of the preliminary estimation results. To compare the performance, typical convolutional neural networks (CNNs) widely adopted in botany research were used. The results showed that the estimated values of the TMSCNet fitted the measurements well, with coefficient of determination (*R*^2^) values of 0.9514, 0.9696, 0.9129, 0.8481, and 0.9495, normalized root mean square error (NRMSE) values of 15.63, 11.80, 11.40, 10.18, and 14.65% and normalized mean squared error (NMSE) value of 0.0826, which was superior to compared methods. Compared with previous studies on the estimation of lettuce traits, the performance of the TMSCNet was still better. The proposed method not only fully considered the correlation between different traits and designed a novel self-correcting structure based on this but also studied more lettuce traits than previous studies. The results indicated that the TMSCNet is an effective method to estimate the lettuce traits and will be extended to the high-throughput situation. Code is available at https://github.com/lxsfight/TMSCNet.git.

## Introduction

Lettuce is a leaf-consuming crop, which has high economic benefits and nutritional value and is planted in a large area all over the world (Adhikari et al., 2019). Lettuce has become a popular vegetable and is often used in salads because of its refreshing and delicious taste, low energy intake, and rich in vitamins and antioxidants (Xu and Mou, 2015). Although lettuce has a rapid growth rate (Grahn et al., 2015) and multiple harvest times, it is sensitive to the growth environment, such as poor adaptability to saline alkali land (Adhikari et al., 2019) Fresh weight (FW), dry weight (DW), height (H), diameter (D), and leaf area (LA) of lettuce are critical indicators to evaluate lettuce (Lati et al., 2013; Teobaldelli et al., 2019; Zhang et al., 2020). Therefore, it is of great significance to monitor the growth traits of lettuce, evaluate the quality of lettuce and determine the harvest time through the growth traits of lettuce.

Plant phenotypic analysis is a significant branch of agriculture (Xiong et al., 2021). It is of great value in determining the best harvest time of crops (Andujar et al., 2016), monitoring crop health, evaluating crop quality and yield, crop management decision-making (Bauer et al., 2019), scientific breeding (Xiong et al., 2017) and so on. The traditional plant phenotype analysis is mainly a manual task, which needs intensive manpower and consumes a lot of time (Casadesus and Villegas, 2014). Yet, the analysis effect is closely related to the operator’s experience requirements, and the analysis effect is often unstable and inaccurate (Zhang et al., 2018). In recent years, non-destructive determination has become a popular and potential method (Jiang et al., 2016). Many researchers have introduced machine learning methods into the non-destructive testing of crops (Greener et al., 2022). Chen et al. built multiple machine learning (ML) methods, including random forest (RF), support vector regression (SVR), and multivariate linear regression (MLR), to estimate the barley biomass (Chen et al., 2018). The study concluded that the RF method estimated barley biomass more accurately than other methods. Fan et al. used the MLR method to estimate the LA of Italian ryegrass and the *R*^2^ value reached 0.79 (Fan et al., 2018). Yoosefzadeh adopted some algorithms such as ensemble bagging (EB), ensemble stacking (E-S), and deep neural network (DNN) to evaluate the soybean yield (Yoosefzadeh, 2021). Tackenberg proposed using linear regression (LR) to estimate growth traits of grass, with the *R*^2^ value of 0.85 (Tackenberg, 2007). Sakamoto et al. (2012) estimated the leaf area index of maize.

Convolutional neural network (CNN) is a typical deep learning method with powerful feature extraction capability, which takes images as input (Ma et al., 2019). Compared with the early machine learning methods, CNN is more stable (Ma et al., 2017) and usually has better performance (Uzal et al., 2018), so it is widely applied to agriculture. Nevertheless, CNN has been widely used in classification tasks, such as plant disease diagnosis (Mohanty et al., 2016; Nachtigall et al., 2016; Ferentinos, 2018), and weed recognition (Dyrmann et al., 2016; Grinblat et al., 2016), and rarely applied to regression tasks of plant phenotype. Ferreira et al. (2017) designed a Deep Convolutional Neural Network to estimate above ground biomass of winter wheat, with the *R*^2^ value of 0.808, and the NRMSE value of 24.95%. Zhang et al. (2020) proposed a convolutional neural network to monitor FW, DW, and LA of lettuce. The result showed that the estimated values had good agreement with measurement results (Zhang et al., 2020).

Although previous studies have achieved good estimation results of lettuce traits, these studies did not consider and make use of the correlation between various traits, and the estimated number of lettuce traits is still insufficient, which will not be conducive to a comprehensive assessment of lettuce growth. For this reason, we developed a three-stage multi-branch self-correcting trait estimation network (TMSCNet) to estimate the fresh weight (FW), dry weight (DW), height (H), diameter (D), and leaf area (LA) of lettuce using RGB images and depth images as input. Then we analyzed the correlation between different traits of lettuce and designed a data preprocessing pipeline. Finally, *R*^2^, NRMSE, and NMSE were used to evaluate the performance of the TMSCNet. To improve the applicability of the TMSCNet, we also discussed the high-throughput case and proposed a processing pipeline.

## Materials and methods

### Dataset and preprocessing

**FIGURE 1:**
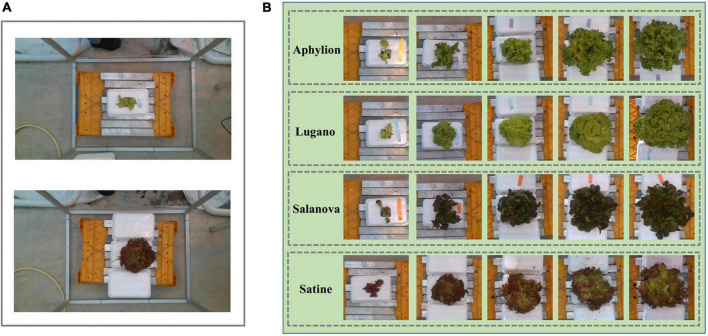
Examples of the RGB images from the public dataset of the 3rd international challenge on autonomous greenhouses. **(A)** The original images, in which the lettuce only accounts for a small proportion. **(B)** The cultivar of Aphylion, Salanova, Satine, and Lugano at different growth stages in the dataset.

The data used in the experiment came from the public dataset of the 3rd international challenge on autonomous greenhouses organized by Tencent and Wageningen University and Research. As one of the participating teams, we participated in this international competition. The public dataset was collected from the lettuce planting laboratory at Wageningen University and research in the Netherlands. All crops were planted in well-controlled greenhouse conditions. Since the enclosure of the greenhouse was highly transparent, the images were collected under natural light. A RealSense D415 depth sensor was used for image collection, which was hung about 0.9 meters above the crop to capture RGB images and depth images. The original pixel resolutions of collected images were 1,920 × 1,080. Those images were stored in PNG format. It is worth noting that the range of image acquisition is about 1 m^2^ of planting area, but the area of the crop only accounts for 5%, as shown in Figure 1A. Four cultivars of lettuce, Aphylion, Salanova, Satine, and Lugano, were grown in a hydroponic growing system, as shown in Figure 1B. The time interval of data acquisition was set to once a week, including six times in total. During this period, 340 images for training and 50 images for validation were obtained (Autonomousgreenhouses, 2018).

Measurements of fresh weight (FW), dry weight (DW), height (H), diameter (D), and leaf area (LA) were conducted simultaneously with image collection, which used a destructive sampling method. The units of these measurements were “gram/plant,” “gram/plant,” “cm,” “cm,” and “cm^2^,” respectively. Among these lettuce traits, height refers to the distance from the first leaf to the highest point of the crop, diameter is the principal diameter of the projection on a horizontal surface, and leaf area is the surface projected on a horizontal surface of all leaves torn from the stem. Fresh weight and dry weight include the weight of root and shoot of lettuce.

**FIGURE 2:**
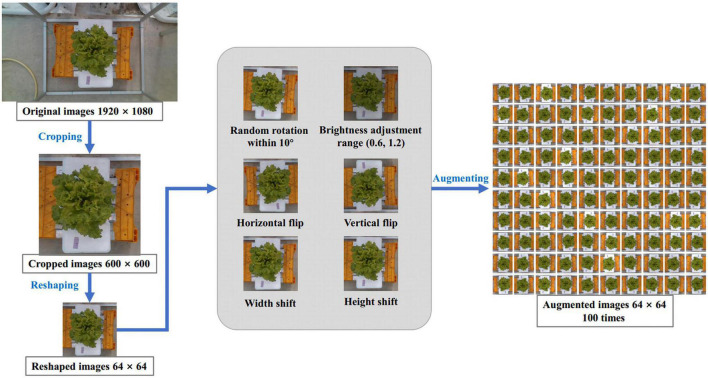
Image process scheme. Input the original image of 1,920 × 1,080 pixels, scale it to 64 × 64 pixels after cropping, and then expand it by 100 times through six ways of random data augmentation.

Since the original RGB images just contained a small proportion of lettuce area, this study firstly automatically cropped images by a 600 × 600 pixels cropping box centered in the center of the image to eliminate the useless part. Then, to improve operating speed, the cropped images were further resized to 64 × 64 pixels. To improve the effect of model training and prevent overfitting, we designed a data augmentation method to enlarge the 340 images of the training dataset. The augmentations method included: rotating the image randomly within 10 degrees, randomly adjusting the brightness of the image in the range of 0.6 to 1.2, flipping the image horizontally and vertically, and shifting the image from width and height by nearest filling mode. Through this series of random augmentation, the training dataset was enlarged by 100 times, resulting in 34,000 RGB images. The complete preprocessing process of the RGB image is demonstrated in Figure 2.

Compared with the RGB image, the depth image looked pure black, because it contained depth information, it was used to estimate the height of lettuce. The preprocessing of depth images was almost the same as that of RGB images, except that the depth images were first converted into the pseudo color map (Liu et al., 2020) before cropping.

### Correlation analysis of growth traits of lettuce

**FIGURE 3:**
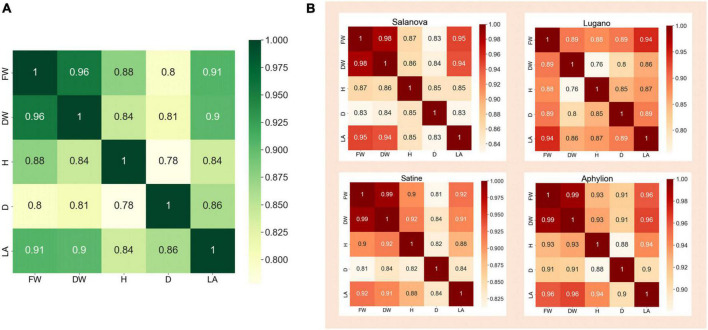
The heat map of correlation analysis of different traits. **(A)** Correlation analysis of all samples. **(B)** Correlation analysis of four lettuce varieties on their traits.

In the dataset, the measurements of FW, DW, H, D, and LA corresponding to 340 lettuce images were organized in a JSON file. It is easy to consider that these traits are not completely independent, but are interrelated. For example, when the fresh weight of lettuce is large, the corresponding dry weight will be heavier; When the diameter of lettuce is longer, the height and leaf area of lettuce will also increase. Therefore, the correlation coefficient was used to perform the correlation analysis on those five traits of lettuce. Figure 3A was the heat map of the correlation analysis of the five traits corresponding to the 340 lettuce images. The number on this figure represented the correlation coefficient of the two traits, and the color depth also reflected the value of the correlation coefficient. It could be seen from the figure that the correlation coefficient between FW and DW was as high as 0.96, the correlation coefficient between FW and LA was 0.91, and the correlation coefficient between H and D was the lowest but still as high as 0.78. This showed that these five traits of lettuce were highly correlated, and it was unreasonable to ignore the correlation between them.

In addition, it could be seen from Figure 1B that different varieties of lettuce had differences in color, shape, growth speed, and so on. Therefore, we further performed the correlation analysis on the five traits of different varieties of lettuce. Figure 3B showed the correlation heat map between those traits of four lettuce varieties, respectively. It could be seen that the correlation coefficient in the heat map of different lettuce varieties was quite different. For example, the correlation coefficient of FW and DW of Aphylion was as high as 0.99, while that of Lugano was only 0.89; The correlation between DW and H of Satine was 0.92, while that of Lugano was only 0.76. This indicated that different lettuce varieties also had an obvious impact on the growth traits of lettuce. Detailed correlation analysis data were illustrated in Tables 1–5.

**TABLE 1:** Correlation between FW and other traits of different lettuce varieties.

Traits	Correlation coefficient				
	Salanova	Lugano	Satine	Aphylion	All
DW	**0.98**	0.89	**0.99**	**0.99**	**0.96**
H	0.87	0.88	0.90	0.93	0.88
D	0.83	0.89	0.81	0.91	0.80
LA	**0.95**	0.94	0.92	**0.96**	0.91

The bold values indicate that the values are greater than or equal to 0.95.

**TABLE 2:** Correlation between DW and other traits of different lettuce varieties.

Traits	Correlation coefficient				
	Salanova	Lugano	Satine	Aphylion	All
FW	**0.98**	0.89	**0.99**	**0.99**	**0.96**
H	0.86	0.76	0.92	0.93	0.84
D	0.84	0.80	0.84	0.91	0.81
LA	0.94	0.86	0.91	**0.96**	0.90

The bold values indicate that the values are greater than or equal to 0.95.

**TABLE 3:** Correlation between H and other traits of different lettuce varieties.

Traits	Correlation coefficient				
	Salanova	Lugano	Satine	Aphylion	All
FW	0.87	0.88	0.90	0.93	0.88
DW	0.86	0.76	0.92	0.93	0.84
D	0.85	0.85	0.82	0.88	0.78
LA	0.85	0.87	0.88	0.94	0.84

**TABLE 4:** Correlation between D and other traits of different lettuce varieties.

Traits	Correlation coefficient				
	Salanova	Lugano	Satine	Aphylion	All
FW	0.83	0.98	0.81	0.91	0.80
DW	0.84	0.80	0.84	0.91	0.81
H	0.85	0.85	0.82	0.88	0.78
LA	0.83	0.89	0.84	0.90	0.86

**TABLE 5:** Correlation between LA and other traits of different lettuce varieties.

Traits	Correlation coefficient				
	Salanova	Lugano	Satine	Aphylion	All
FW	**0.95**	0.94	0.92	**0.96**	0.91
DW	0.94	0.86	0.91	**0.96**	0.90
H	0.85	0.87	0.88	0.94	0.84
D	0.83	0.89	0.84	0.90	0.86

The bold values indicate that the values are greater than or equal to 0.95.

### A three-stage multi-branch self-correcting trait estimation network

#### Construction of the three-stage multi-branch self-correcting trait estimation network

**FIGURE 4:**
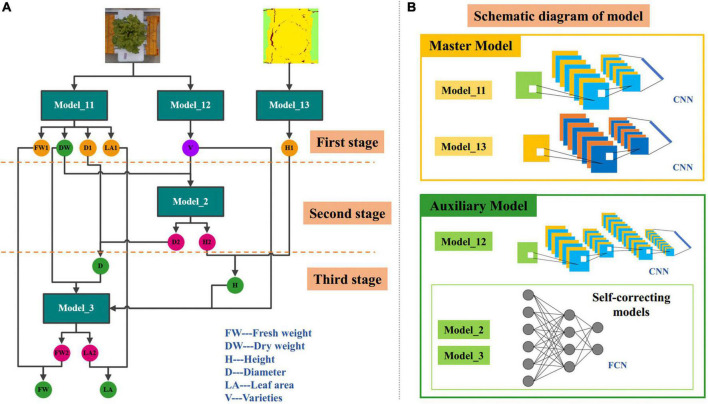
Architecture of the three-stage multi-branch self-correcting network (TMSCNet). **(A)** The principle of TMSCNet. **(B)** Schematic diagram of the models in the TMSCNet.

Accurate and efficient traits estimation is the crux to precise control and decision-making of lettuce crops and a precondition for quantifying plant traits. The estimation of lettuce traits is a regression task, whose goal is to estimate the five traits of FW, DW, H, D, and LA through images. Compared with the RGB image, the depth image was more suitable for lettuce height estimation because it contained depth information. Therefore, we proposed two master models. One master model used RGB image as input to estimate LW, DW, D, and LA of lettuce; The other master model used the pseudo color image of depth image conversion as input to estimate H. After the analysis, we found that the five lettuce traits were highly correlated with each other, and were affected by the variety of lettuce, so we further proposed three auxiliary models. One of the three auxiliary models was used to predict the variety of lettuce through RGB images, and the function of the other two auxiliary models was to cooperate with the classification auxiliary model of variety to automatically correct the estimation results of master models. The above five models constituted a three-stage multi-branch self-correcting trait estimation network (TMSCNet), and its overall structure was shown in Figures 4A,B. Model_11 and Model_13 were two master models, which were located in the first stage of the network. In the first stage, there still had a Model_12 for predicting lettuce varieties; The second stage and the third stage of the network each had an auxiliary self-correcting model, which were named Model_2 and Model_3.

The operating principle of TMSCNet was as follows: 340 RGB images and depth images were preprocessed and then inputted into the two regression master models in the first stage to get the preliminary five growth traits; Then, the prediction varieties of lettuce obtained by the classification model and DW estimated by the Model_11 were used as the input of the regression model in the second stage to generate new values of H and D, and they corrected the values of preliminary H and D obtained by the Model_11 in the first stage; Finally, DW obtained in the first stage, the variety of lettuce, and the corrected values of D and H obtained in the second stage were fed into the regression model of the third stage to calculate new values of FW and LA, and they were further used to correct the values of FW and LA obtained in the first stage. So far, all lettuce traits have been estimated and automatically corrected.

It is worth noting that the second and third stage models take DW obtained in the first stage as the input and take it as the final DW of lettuce. The reason is that DW is the most accurate prediction among all traits, which may not be intuitive, but it is the conclusion obtained through our experiments.

#### Master model

**FIGURE 5:**
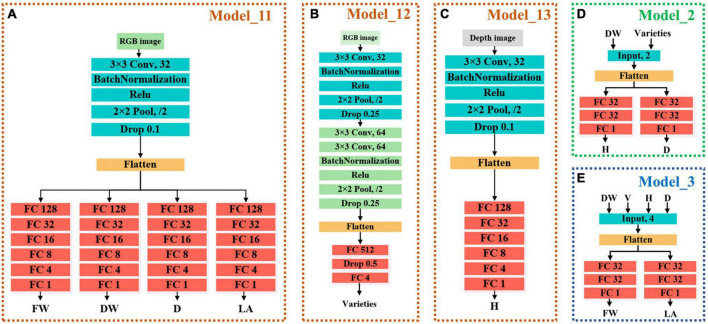
Structure of each model. **(A)** Model_11 of the master models for estimating FW, DW, D, and LA. **(B)** Model_12 of the auxiliary models for predicting lettuce varieties. **(C)** Model_12 of the master models for estimating H. **(D)** Model_2 of self-correcting models of the auxiliary models for automatically adjusting H and D, which are located in the second stages of TMSCNet. **(E)** Model_3 of self-correcting models of the auxiliary models for automatically adjusting FW and LA, which are located in the third stages of TMSCNet.

The main model included Model_11 for estimating FW, DW, D, and LA, and Model_13 for estimating H. Model_11 was a convolutional neural network (CNN) model with single input and four outputs. The input of Model_11 was RGB images of lettuce with a size of 64 × 64 × 3 (width × height × channel). Model_11 has one convolutional layer to extract features that adopted kernels with a size of 3 × 3 and the number of kernels was 32. The batch normalization layer was used to prevent the elimination of gradient. The max pooling function was adopted in the pooling layer, which had a pooling size of 2 × 2 and a stride of 2 to reduce the size of feature maps. The rate of the dropout layer was 0.1. After passing through the flatten layer, the dimension of the feature was reduced so that it was sent to the fully connected network. The fully connected layers included four branches, each branch had five fully connected layers, and the number of neurons was 128, 32, 16, 8, and 4. Each branch outputted one result for estimating FW, DW, D, and LA, respectively. The structure of Model_11 was shown in Figure 5A.

Model_13 had the same structure and network parameters as Model_11, except that its input was the pseudo color image obtained by depth image conversion, and there was only one trunk in the full connection layer to estimate H, as shown in Figure 5B.

#### Auxiliary model

The proposed TMSCNet included three auxiliary models, which were located in three stages to assist the master model to complete the automatic correction of estimation traits. These three auxiliary models included a classification model for predicting lettuce varieties and two self-correcting regression models.

The input of Model_12 was also RGB images with a size of 64 × 64 × 3. The structure of Model_12 was shown in Figure 5C, with three convolutional layers, which adopt kernels with the size of 3 × 3. The number of kernels in the three convolutional layers was 32, 64, and 64. The max pooling layer had a pooling size of 2 × 2 and a stride of 2 too. The full connection layer had only one hidden layer with 512 units, followed by a dropout layer with a rate of 0.5. The output layer included four units, which were used to predict the four varieties of lettuce.

The two self-correcting models located in the second and third stages of TMSCNet were two deep neural networks (DNN), whose input data was not images, but values read from label files. Their model structure was shown in Figures 5D,E. Model_2 took DW and lettuce varieties as input. After passing through a flatten layer, the data was divided into two branches, each branch had two full connection layers with 32 units and finally outputted the estimated values of H and D. Model_3 had the same structure and parameter settings as Model_2. Its inputs were DW, H, D, and lettuce varieties, and its outputs were estimated values of FW and LA.

The output results of Model_2 and Model_3 were not the final lettuce estimated values but were only used to automatically correct the output of master models. The modified calculation formula of Model_2 was shown in Equation 1, where k_*H*_ and k_*D*_ were weight values for adjustment parameters, and the footmark indicates the stage of traits acquisition. In other words, y_*H*1_ and y_*D*1_ were the results of master models estimation, y_*H*2_ and y_*D*2_ were the results of Model_2 estimation, and the revised new H and D estimates were obtained through weighted calculation.


(1) $$\begin{array}{c} {\text{y}}_{\text{H}}={\text{y}}_{\mathrm{H1}}\times {\text{k}}_{H}+{\text{y}}_{\mathrm{H2}}\times \left(1-{\text{k}}_{\text{H}}\right)\\ {\text{y}}_{\text{D}}={\text{y}}_{\mathrm{D1}}\times {\text{k}}_{\text{D}}+{\text{y}}_{\mathrm{D2}}\times \left(1-{\text{k}}_{\text{D}}\right) \end{array}$$

Similarly, the correction formula of Model_3 was demonstrated in Eq. 2, which was used to correct FW and LA of Model_11 estimation by the two results estimated by Model_3.


(2) $$\begin{array}{c} {\text{y}}_{FW}={\text{y}}_{FW1}\times {\text{k}}_{FW}+{\text{y}}_{FW3}\times \left(1-{\text{k}}_{FW}\right)\\ {\text{y}}_{LA}={\text{y}}_{LA1}\times {\text{k}}_{LA}+{\text{y}}_{LA3}\times \left(1-{\text{k}}_{LA}\right) \end{array}$$

By analyzing the correlation between the traits of lettuce and the actual experimental results, it was determined that k_*H*_, k_*D*_, k_*FW*_, and k_*LA*_ were 0.59, 0.98, 0.60, and 0.96, respectively.

## Results

### Experimental setup

In this study, the construction of the TMSCNet and image preprocessing were implemented using TensorFlow 2.4 under the Windows 10 operating system. The configuration of the computer was as follows: Intel(R) Core (TM) i7-10875H CPU @ 2.30 GHz, 16.0 GB RAM, NVIDIA GeForce GTX1650.

We adopted the normalized root mean square error (NRMSE) and the coefficient of determination (*R*^2^) to evaluate the performances of a single trait and used the normalized mean squared error (NMSE) to comprehensively evaluate the performance of five lettuce traits, as shown in Eq. 3.


(3) $$\begin{array}{c} NRMSE=\frac{\sqrt{\sum_{i=0}^{n}{\left(y_{i}-{\hat{y}}_{i}\right)}^{2}}}{\sqrt{\sum_{i=0}^{n}{\left(y_{i}\right)}^{2}}}\\ R^{2}=1-\frac{\sum_{i=0}^{n}{\left(y_{i}-{\hat{y}}_{i}\right)}^{2}}{\sum_{i=0}^{n}{\left(y_{i}-{\bar{y}}_{i}\right)}^{2}}\\ \mathrm{NMSE}=\sum_{j=0}^{m}\frac{\sum_{i=0}^{n}{\left(y_{ij}-{\hat{y}}_{ij}\right)}^{2}}{\sum_{i=0}^{n}{\left(y_{ij}\right)}^{2}} \end{array}$$

Where y referred to the measured value, *_ŷ_* was the value estimated by the network, *_ȳ_* was the average value of the measured value, n was the number of samples, and m was the number of lettuce traits.

In this study, the five models of the TMSCNet used the Adam optimizer with the learning rate of 0.001 to optimize the model weights and split 340 training samples into the training dataset and test dataset according to the ratio of 8:2. The batch size of Model_11, Model_12, Model_13, Model_2, and Model_3 was set to 170, 32, 500, 16, and 16, respectively. The number of epochs was set to 50, 50, 50, 200, and 200. Besides, the loss function of four regression models was the mean square error (MSE), and the classification model of lettuce varieties used cross entropy as the loss function.

### Training and estimation of the three-stage multi-branch self-correcting trait estimation network

**FIGURE 6:**
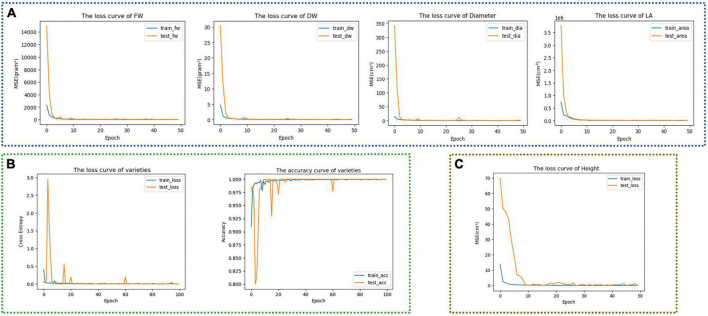
Training curves of different models. **(A)** Training curves of FW, DW, D, and LA of Model_11. **(B)** Training curve and accuracy curve of lettuce varieties of Model_12. **(C)** Training curve of H of Model_13.

Trained each model of the TMSCNet according to the configuration in the experimental setup. After 50 epochs, the loss curves of Model_11 were shown in Figure 6A. It could be seen that those four curves dropped rapidly at the beginning, and then the curves were stable without significant fluctuation. In addition, the training curves were very close to the testing curve, indicating that the model had no prominent overfitting and underfitting problems. Figure 6B showed the training curve of Model_12, the left was the loss curve, and the right was the accuracy curve. Although there were some fluctuations in the curve of the test set, it still overlapped with the training curve as a whole, and after the 80th epoch, the accuracy rate exceeded 99%, indicating that the trained model could accurately predict lettuce varieties. Model_13 was only used to estimate H. It could be seen from the loss function curve in Figure 6C that after the 10th epoch, MSE was almost reduced to 0, and the curve was relatively stable. Then, two self-correcting models in the second and third stages were trained.

**FIGURE 7:**
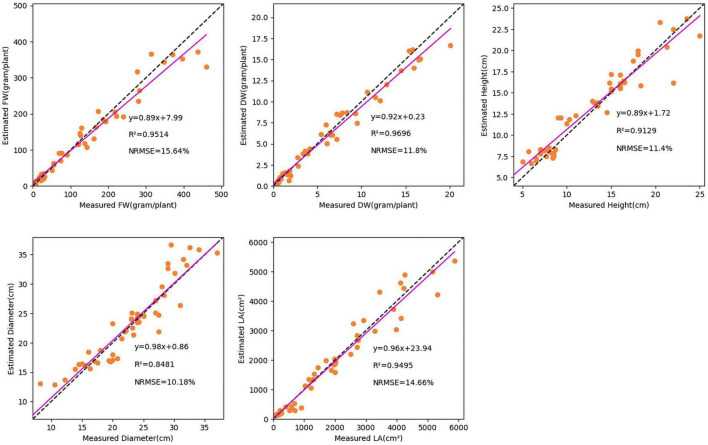
Estimation result of lettuce traits based on the TMSCNet. Five subgraphs show the estimated results of FW, DW, H, D, and LA, respectively.

To evaluate the performance, 50 validation images were used to test the TMSCNet that has completed the training. Figure 7 showed the results on the validation set. The abscissa represented the measured value, the ordinate represented the estimated value, and the scatter points reflected the prediction results. The magenta straight line was the curve of the equation between the measured value and the estimated value fitted by the least square method. The lower right corner of each subgraph showed the evaluation score and fitting curve equation of every single trait. Where *R*^2^ was in the range of 0–1, the larger its value was, the better the estimation effect was. While the smaller the NRMSE value was, the better the network performance was. The fitting line also reflected the performance of the network. When it was closer to the diagonal represented by the dotted line, the better the evaluation effect was. For the five growth traits of FW, DW, H, D, and LA, the TMSCNet had *R*^2^ values equal to 0.9514, 0.9696, 0.9129, 0.8481, and 0.9495, respectively, and NRMSE values equal to 15.64, 11.80, 11.40, 10.18, and 14.66%, respectively. The results reflected that the proposed lettuce estimation network had a great performance and estimation ability.

**FIGURE 8:**
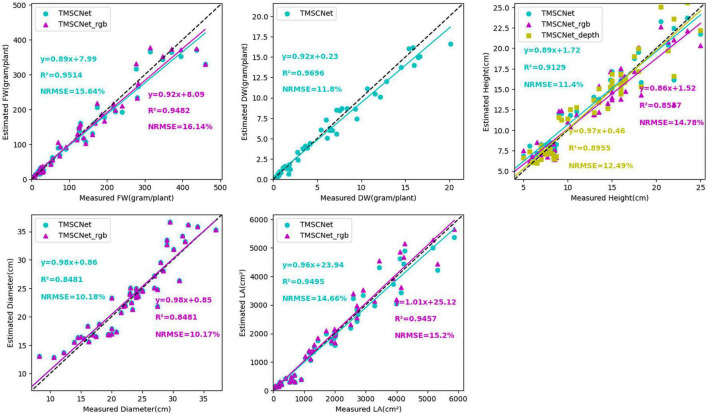
Estimation result of lettuce traits based on the Model_11, Model_13, and TMSCNet.

In order to verify that the proposed multi-stage self-correcting network was better than the evaluation effect of using only the master model, the results of evaluating lettuce traits using only Model_11, combined Model_11 and Model_13, and TMSCNet using five models were compared. The comparison results were shown in Figure 8, where cyan represented TMSCNet, magenta represented Model_11 of TMSCNet, and yellow represented Model_13 of TMSCNet. From the comparison results, it could be seen that the result of using the self-correction model of the five models was indeed better than using only the master model. In the subgraph of FW, the *R*^2^ value of TMSCNet increased by 0.0032, and the value of NRMSE decreased by 0.5%, while in the subgraph of LA, the *R*^2^ value of TMSCNet increased by 0.048 and the value of NRMSE decreased by 0.54%. From the subgraph of H, the effect of evaluation with depth image was indeed better than that with RGB image, which was also the reason why Model_13 was used in the master model. Compared with them, TMSCNet further improved the estimation result of H, the *R*^2^ value was equal to 0.9129, which was 0.0174 higher than Model_13, NMSE value was equal to 11.4%, which was 1.09% lower than Model_13. Since the DW estimated by Model_11 was its final estimation result, there was no other comparison curve in the sub-graph of DW. In addition, from the estimation results of DW, it could be seen that among the five traits, its estimation result was the most accurate, which was the reason why the DW estimated by Model_11 was used as the final result without correction, and also the reason why DW was used as the input of Model_2 and Model_3 to correct other traits.

### Comparison of the results with the classical convolutional neural networks methods

At present, many classical deep learning networks had been proposed. These networks had a more complex network structure and great operation effect and had been widely used in botany research. To verify the superiority of the proposed TMSCNet, we did comparative experiments to compare the performance of the model on 50 validation datasets under basically the same conditions. The models used for comparative experiments were VGG16 (Simonyan and Zisserman, 2014), Xception (Chollet and Ieee, 2017), Resnet50 (He et al., 2016), and Densenet121 (Huang et al., 2017). VGG model had two convolution and three convolution modules, which improves the feature extraction ability of the convolution layer. Xception network adopted deep separable convolution. Resnet model introduced the idea of residual, which effectively alleviated the problem of gradient disappearance caused by the deepening of network layers. Densenet network adopted an intensive connection mode to reduce information loss.

Table 6 demonstrated the *R*^2^ values and NRMSE values of five traits of lettuce estimated by different methods. It was known from this table that Resnet50 and Densenet121 performed well, and Densenet121 had the best estimation effect on D, while the *R*^2^ values and NMSE values of FW, DW, H, and LA estimated by the proposed TMSCNet were better than other methods.

**TABLE 6:** *R*^2^ values and NRMSE values of the estimated lettuce traits by different classical CNNs and TMSCNet.

Method	Fresh weight		Dry weight		Height	
	*R* ^2^	NRMSE	*R* ^2^	NRMSE	*R* ^2^	NRMSE
VGG16	0.8208	30.02%	0.8621	25.15%	0.7265	20.20%
Xception	0.8135	30.63%	0.8875	22.71%	0.6824	21.77%
Resnet50	0.9123	21.01%	0.9433	16.13%	0.7791	18.16%
Densenet121	0.9243	19.52%	0.9540	14.53%	0.8055	17.04%
TMSCNet	**0.9514**	**15.63%**	**0.9696**	**11.80%**	**0.9129**	**11.40%**

**Method**	**Diameter**		**Leaf area**			
	***R* ^2^**	**NRMSE**	***R* ^2^**	**NRMSE**		

VGG16	0.7928	11.88%	0.9333	16.85%		
Xception	0.7275	13.63%	0.8894	21.69%		
Resnet50	0.7693	12.54%	0.9329	16.89%		
Densenet121	**0.8498**	**10.12%**	0.9188	18.59%		
TMSCNet	0.8481	10.18%	**0.9495**	**14.65%**		

The bold values indicate the best results.

**FIGURE 9:**
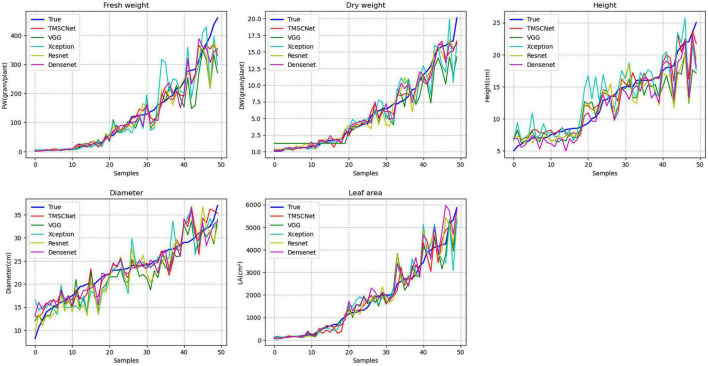
Comparison curve of evaluation results based on different classical convolutional neural networks (CNNs) and TMSCNet.

Normalized mean squared error was used to further comprehensively evaluate each method. The calculated NMSE values of VGG16, Xception, Resnet50, Densenet121, and TMSCNet were 0.2367, 0.2584, 0.1474, 0.1330, and 0.0826, respectively, as shown in Table 7. In the table TMSCNet_1 and TMSCNet_2 referred to only using Model_11 and combined Model_11 and Model_13. To more intuitively displayed the estimated results of several methods, the estimated value and measured values of five traits were drawn, in which the measured values were arranged in the order from small to large, and the estimated values also were arranged in this way, as shown in Figure 9. In the figure, the measured value was represented by blue lines, the proposed TMSCNet was represented by red lines, and VGG16, Xception, Resnet50, and Densenet121 were represented by green lines, cyan lines, yellow lines, and magenta lines, respectively. It could be seen that the estimated results fluctuated around the measured values, in which the curve of Xception deviates from the measured value most obviously, and the red line was closest to the measured value curve. In comparison with the classical CNN methods, the proposed TMSCNet showed superior estimation performances in estimating the five growth traits of lettuce.

**TABLE 7:** NMSE values of the estimated lettuce traits by different classical CNNs and TMSCNet.

	Method	NMSE
The classical models	VGG16	0.2367
	Xception	0.2584
	Resnet50	0.1474
	Densenet121	0.1330
The proposed model	TMSCNet_1	0.0952
	TMSCNet_2	0.0896
	TMSCNet	**0.0826**

The bold values indicate the best results.

## Discussion

### Preprocessing method for estimating H

**FIGURE 10:**
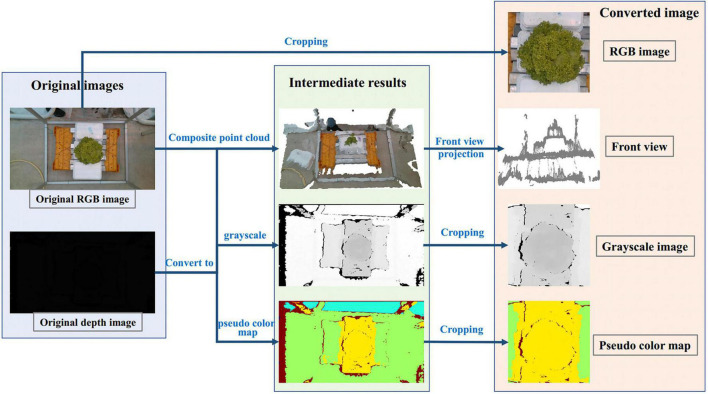
Four different processing methods for estimating H. The first one is to estimate H with the cropped RGB image; The second is the front view estimation of H obtained from the point cloud projection synthesized by RGB and depth images; The third is to convert the depth image into the grayscale image; The fourth is to convert depth image into the pseudo color image. Among them, the fourth method to convert the depth image into the pseudo color image is the data processing method of TMSCNet for estimating H.

For the H of lettuce, we proposed four preprocessing methods in the early stage, as shown in Figure 10. The first method was to use RGB image as input; The second method was to use RGB image and depth image combined with camera internal parameters to convert 3D point cloud, and then project them along the direction of the main view to get a front view that can reflect the height; The third method was to convert the depth image into the gray-scale map after standardization; The fourth method was to use OpenCV to convert depth image into the pseudo color map. Those four preprocessing methods had a similar process, which was resized from 600 × 600 pixels to 64 × 64 pixels and augmented 100 times.

To select the most suitable data preprocessing method for lettuce height estimation, the four methods were evaluated by the *R*^2^ values and NRMSE values on the validation dataset containing 50 samples. Table 8 showed the evaluation scores of different data preprocessing methods for H. The *R*^2^ values of methods 1 to 4 were 08537, 0.6337, 0.8130, and 0.8912, respectively, while the NRMSE values were 14.78, 23.38, 16.71, and 12.74%, respectively. Among them, the best way to estimate the height of lettuce was to convert the depth image into a pseudo color map.

**TABLE 8:** Results of the estimated H by four date processing methods.

	Method	Fitted formula	*R* ^2^	NRMSE
H	RGB	*y* = 0.8613×x+1.5161	0.8537	14.78%
	Depth_Front_view	*y* = 0.6442×x+2.2012	0.6337	23.38%
	Depth_grayscale	*y* = 0.9164×x+2.1408	0.8130	16.71%
	Depth_pseudo_color	*y* = 0.9317×x+1.6150	**0.8912**	**12.74%**

The bold values indicate the best results.

**FIGURE 11:**
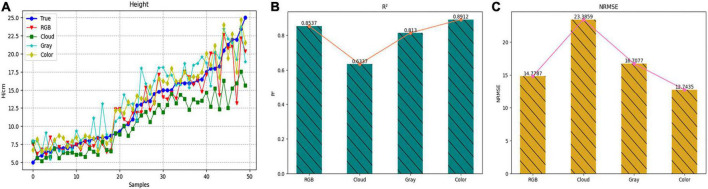
Estimation result of H based on four different processing methods. **(A)** Comparison curve of evaluation results. **(B)** Histogram and line chart of *R*^2^ value of evaluation results. **(C)** Histogram and line chart of NRMSE value of evaluation results.

To more intuitively displayed the estimated results of the height value of each method, Figure 11A was drawn, in which the blue line represented the measured value, the red line represented the method of estimating the height with the RGB image, and the green line, cyan line and yellow line, respectively, represented the method of converting the depth image into front view, gray map, and pseudo color map. It could also be seen from this figure that the evaluation curve of the method of converting the depth map into a pseudo color map was the closest to the measurement result curve, so the estimation effect was the best. Figures 11B,C showed the evaluation scores of these four methods more intuitively in the form of a histogram. Therefore, the Model_13 of TMSCNet used the pseudo color map converted from the depth image as input to train the model and to estimate the H.

### The selection of self-correcting models of the TMSCNet

Since the input of the auxiliary models in the second and third stages of the TMSCNet was the numerical data rather than the image, the classical machine learning regression algorithm and fully connected network (FCN) were considered. There were many classical regression models in machine learning, such as Linear Regression (Austin and Steyerberg, 2015), Support Vector Regressor (Cortes and Vapnik, 1995), K-Nearest Neighbors Regressor (Song et al., 2017), Decision Tree Regressor (Luo et al., 2021), Random Forest Regressor (Ding and Bar-Joseph, 2017), AdaBoost Regressor (Chen et al., 2019), and Bagging Regressor (Dal Molin Ribeiro and Coelho, 2020). For selecting more suitable self-correcting models, we first conducted comparative experiments on the classical machine learning regression algorithm to select the best performing method, and then implement comparative experiments on the optimized classical machine learning algorithm and fully connected network to determine the method of the auxiliary model in the second and third stages of the TMSCNet.

Because the auxiliary models in the second stage and the third stage had similar structures, here we took Model_3 in the third stage of the TMSCNet as an example to carry out the comparative experiment of classical machine learning regression algorithms. The input data of the comparison experiment went through the same operation: extracted the information from the label file, next standardized the data, and then randomly shuffled the data and fed them into the different models. The effect of different algorithms was evaluated by the mean square error (MSE), as shown in Eq. 4.


(4) $$\text{MSE}=\frac{1}{n}\sum_{i=1}^{m}{\left(y_{i}-{\hat{y}}_{i}\right)}^{2}$$

In the regression model of the third stage, the input data were the values of DW, variety, H, and D, and the output results were the values of FW and LA. A 340 samples were used to train each model, and 50 verification samples were estimated. The results of each model were shown in Table 9. It could be seen from this table that the MSE value of the Decision Tree Regressor (DTR) algorithm for the leaf area was quite large, indicating that the effect of this algorithm was unsuited; The MSE values of Support Vector Regressor (SVR) and Random Forest Regressor (RFR) were low, indicating that the two algorithms had better estimation effect, and the performance of SVR was best. Therefore, the SVR algorithm was selected as an alternative algorithm applied in the second and third stages of the TMSCNet.

**TABLE 9:** The MSE results of different machine learning algorithms in the third stage.

Order number	Method	MSE	
		Fresh weight	Leaf area
1	Linear regression	1187.5472	156874.7535
2	Decision tree regressor	1171.7190	706175.2030
3	Support vector regressor	656.8072	116738.7902
4	K neighbors regressor	1094.8050	123418.9811
5	Random forest regressor	842.7343	181768.7793
6	Adaboost regressor	885.7827	199491.6020
7	Bagging regressor	903.6413	210840.3104

**FIGURE 12:**
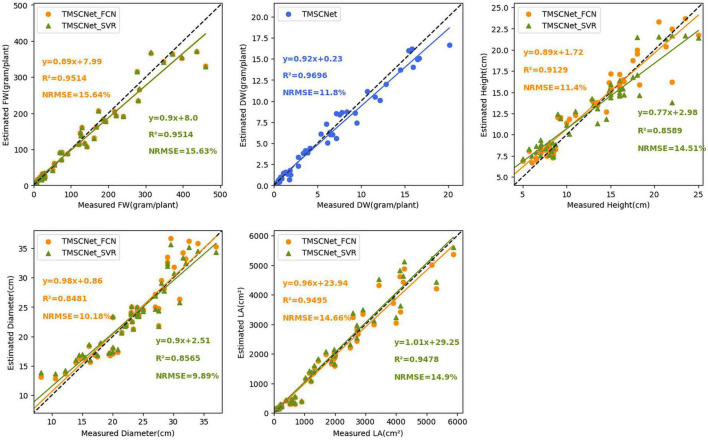
Estimation result of lettuce traits based on TMSCNet with different self-correcting models. TMSCNet_SVR uses SVR as self-correcting models. TMSCNet_FCN adopts FCN as self-correcting models. Among them, the network takes FCN as the self-correcting model.

Figure 12 showed the details of the evaluation on the validation dataset. From the *R*^2^ value and NRMSE value calculated in the figure, it could be seen that H and LA estimated with FCN as the self-correcting model were slightly better than those estimated with SVR, and for D, the estimation effect of SVR was better. To finally determine the method used as self-correcting models of the TMSCNet, the SVR algorithm was compared with the FCN method, and 50 validation samples were estimated to calculate NMSE values. The NMSE value obtained by using SVR as the self-correcting model algorithm was 0.091425, while the NMSE value obtained by using FCN as the self-correcting model algorithm was 0.082658. From the results, the NMSE value obtained by the FCN method was smaller, which showed that the FCN method had a better automatic correction effect. So, the FCN algorithm was determined as self-correcting model of the TMSCNet.

### High-throughput detection and evaluation pipeline

**FIGURE 13:**
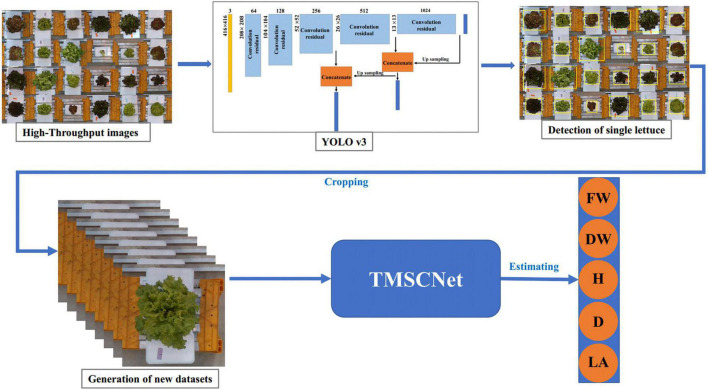
The process of lettuce traits estimation in the high-throughput situation. The lettuce in the high-throughput image is detected based on YOLO v3, and then the detected lettuce is cropped out from the high-throughput image to form new datasets containing only single lettuce. Finally, after data processing, it is inputted to the TMSCNet to estimate the lettuce traits.

In the actual lettuce planting process, to ensure the yield, lettuce was intensively planted in the greenhouse, so the images collected by the camera were high-throughput, namely, the images contain many lettuces rather than just single lettuce. To improve the generality of the proposed TMSCNet, we discussed a high-throughput estimation of the lettuce traits method based on YOLO v3. It included two parts of lettuce detection and traits estimation of lettuce. Lettuce detection was used to identify the position of lettuces and cropped those detected lettuce from the high-throughput image into images of single lettuce. Then the processed image was used to estimate the traits of lettuce using TMSCNet. The process was shown in Figure 13.

### Advantages, limitations and future work

Estimating traits to monitor crop growth is critical for crop production and management (Tudela et al., 2017). In recent years, non-destructive determination has become an efficient and potential method. In the existing studies of lettuce traits estimation, we proposed the method still has advantages. Table 10 shows the details. Zhang et al. (2020) proposed a convolutional neural network to monitor LW, DW, and LA of lettuce, which *R*^2^ values were 08938, 0.8910, and 0.9156, respectively, while the NRMSE values were 26.00, 22.07, and 19.94%, respectively. Mortensen et al. (2018) adopted 3D point clouds to estimate FW, which *R*^2^ values were 0.9400. For FW and DW, the *R*^2^ values of Hu et al. (2018) were 0.9281 and 0.8402. Concepcion et al. (2020a,b) used Gaussian processing regression (GPR) to estimating FW and H with *R*^2^ values of 0.7939 and 0.7662. In addition, the study also carried out comparative experiments with SVR and RF and gave the evaluation scores of these two methods. Compared with these studies, our proposed TMSCNet not only estimates more lettuce traits but also has better evaluation scores than other studies. In addition, TMSCNet fully considers the correlation of various traits and the differences in lettuce varieties and designs an automatic correction network structure based on this, which further improves the accuracy of lettuce trait estimation.

**TABLE 10:** Results of the estimated lettuce traits by previous studies and TMSCNet.

Method		Fresh weight		Dry weight		Leaf area	
		*R* ^2^	NRMSE	*R* ^2^	NRMSE	*R* ^2^	NRMSE
Zhang et al., 2020		0.8938	26.00%	0.8910	22.07%	0.9156	19.94%
Mortensen et al., 2018		0.9400	/	/	/	/	/
Hu et al., 2018		0.9281	/	0.8402	/	/	/
TMSCNet (ours)		**0.9514**	**15.63%**	**0.9696**	**11.80%**	**0.9495**	**14.65%**

**Method**		**Fresh weight**		**Height**			
		***R* ^2^**	**NRMSE**	***R* ^2^**	**NRMSE**		

Concepcion et al., 2020a,b	SVM	0.5683	/	0.7656	/		
	RF	0.7567	/	0.7164	/		
	GPR	0.7939	/	0.7662	/		
TMSCNet (ours)		**0.9514**	**15.63%**	**0.9129**	**11.40%**		

The bold values indicate the best results.

In addition, there are some studies on other lettuce traits, which have similar names to the lettuce traits we studied. Lauguico et al. (2020) adopted DarkNet-53 to predict modeling area and equivdiameter with *R*^2^ values of 0.99 and 0.98. Since the modeling area and equivdiameter of this study had different concepts from the LA and D we evaluated, and the measurements used in this study were measured by image calculation rather than measured by destructive experiments, it was incompatible with our research. Concepcion et al. (2020b) adopted a new genetic algorithm (GA) to compensate peak wavelength sensitivities of the generic camera, and then used a convolutional neural network of MobileNetV2 to estimate canopy area with *R*^2^ values of 0.9805. However, the canopy area was the area measured by the binary image, which was different from the LA measured by the destructive experiment. Therefore, this study was still not comparable with our research.

Although the proposed method has been proven to be superior and accurate, there are still some limitations that we need to be considered. The first limitation is that lettuce at different growth stages has different characteristics, yet the time sequence is not taken into account in traits estimation. The second limitation is that in these five traits, the *R*^2^ value of D is 0.8481, which still has a large deviation from other traits.

In the future work, we will first consider the estimation of lettuce traits under the high-throughput situation, and then intend to deploy TMSCNet to the controller to realize real-time monitoring and automatic control of lettuce. In addition, we also plan to apply this method to other crops.

## Conclusion

In this study, we developed a three-stage multi-branch self-correcting trait estimation network (TMSCNet) to estimate the fresh weight (FW), dry weight (DW), height (H), diameter (D), and leaf area (LA) of lettuce based on RGB and depth images. The TMSCNet was composed of two master models (Model_11 and Model_13) and three auxiliary models (Model_12, Model_2, and Model_3), which were distributed in three stages. Firstly, we carried out the correlation analysis on the five traits of lettuce and found that the five traits were highly correlated and the variety of lettuce also had a great impact on these traits. This analysis provided a basis for the proposed TMSCNet. An appropriate data processing method was the key to model training. Through cutting, resizing and data augmentation, the data preprocessing of RGB images and depth images effectively improved the operation efficiency and estimation effect of the models. Then, we trained the master models and auxiliary models of the proposed TMSCNet and estimated them on the verification dataset, then calculated the *R*^2^ value and NRMSE value of each trait and comprehensively evaluated network performance by NMSE value. Finally, we carried out comparative experiments, and compare the proposed TMSCNet with the classical CNNs on lettuce trait estimation. The proposed network was superior to other models, and the estimated values of lettuce traits were in good agreement with the measured values with *R*^2^ value of about 0.92, NRMSE value of about 13%, and NMSE value of 0.0826. Moreover, the performance of the proposed method was also superior to that of the previous studies to estimate lettuce traits. It indicated that the proposed TMSCNet is an effective and accurate tool for the estimation of lettuce traits and has the feasibility of extending to high-throughput data. Furthermore, this method can be applied to the estimation of other crops.

## Data availability statement

The original contributions presented in this study are included in the article/supplementary material, further inquiries can be directed to the corresponding author.

## Author contributions

QZ and YW organized this research and revised the manuscript. XZ designed the three-stage multi-branch TMSCNet and drafted the manuscript. XZ and XL developed the experiments. All authors contributed to the article and approved the submitted version.

## References

[B1] Adhikari N. D. Simko I. Mou B. (2019). Phenomic and physiological analysis of salinity effects on lettuce. *Sensors* 19:21. 10.3390/s19214814 31694293 PMC6864466

[B2] Andujar D. Ribeiro A. Fernandez-Quintanilla C. Dorado J. (2016). Using depth cameras to extract structural parameters to assess the growth state and yield of cauliflower crops. *Comput. Electron. Agric.* 122 67–73. 10.1016/j.compag.2016.01.018

[B3] Austin P. C. Steyerberg E. W. (2015). The number of subjects per variable is required in linear regression analyses. *J. Clin. Epidem.* 68 627–636. 10.1016/j.jclinepi.2014.12.014 25704724

[B4] Autonomousgreenhouses (2018). *Autonomous greenhouses international challenge 3rd.* Available online at: http://www.autonomousgreenhouses.com/ (accessed February 6, 2018).

[B5] Bauer A. Bostrom A. G. Ball J. Applegate C. Cheng T. Laycock S. (2019). Combining computer vision and deep learning to enable ultra-scale aerial phenotyping and precision agriculture: A case study of lettuce production. *Hortic. Res.* 6:70. 10.1038/s41438-019-0151-5 31231528 PMC6544649

[B6] Casadesus J. Villegas D. (2014). Conventional digital cameras as a tool for assessing leaf area index and biomass for cereal breeding. *J. Integr. Plant Bio.* 56 7–14. 10.1111/jipb.12117 24330531

[B7] Chen D. Shi R. Pape J.-M. Neumann K. Arend D. Graner A. (2018). Predicting plant biomass accumulation from image-derived parameters. *Gigascience* 7 1–13. 10.1093/gigascience/giy001 29346559 PMC5827348

[B8] Chen J. Q. Chen H. Y. Dai W. j Lv Q. J. Chen C. Y. C. (2019). Artificial intelligence approach to find lead compounds for treating tumors. *J. Phys. Chem. Lett.* 10 4382–4400. 10.1021/acs.jpclett.9b01426 31304749

[B9] Chollet F. Ieee. (2017). “Xception: Deep learning with depth wise separable convolutions,” in *Proceedings of the IEEE Conference on Computer Vision and Pattern Recognition*, (Honolulu). 10.1109/CVPR.2017.195

[B10] Concepcion R. Lauguico S. Tobias R. R. Dadios E. Bandala A. Sybingco E. (2020a). “Genetic algorithm-based visible band tetrahedron greenness index modeling for lettuce biophysical signature estimation,” in *Proceedings of the IEEE REGION 10 CONFERENCE*, (Osaka). 10.1109/TENCON50793.2020.9293916

[B11] Concepcion R. Lauguico S. Tobias R. R. Dadios E. Bandala A. Sybingco E. (2020b). “Estimation of photosynthetic growth signature at the canopy scale using new genetic algorithm-modified visible band triangular greenness index,” in *Proceedings of the International Conference on Advanced Robotics and Intelligent Systems*, (Taipei). 10.1109/ARIS50834.2020.9205787

[B12] Cortes C. Vapnik V. J. M. l (1995). Support-vector networks. *Mach. Learn.* 20 273–297. 10.1023/A:1022627411411

[B13] Dal Molin Ribeiro M. H. Coelho L. d. S (2020). Ensemble approach based on bagging, boosting and stacking for short-term prediction in agribusiness time series. *Appl. Soft. Comput.* 86:105837. 10.1016/j.asoc.2019.105837

[B14] Ding J. Bar-Joseph Z. (2017). MethRaFo: MeDIP-seq methylation estimate using a random forest regressor. *Bioinformatics* 33 3477–3479. 10.1093/bioinformatics/btx449 29036558 PMC5860172

[B15] Dyrmann M. Karstoft H. Midtiby H. S. (2016). Plant species classification using deep convolutional neural network. *Biosyst. Eng.* 151 72–80. 10.1016/j.biosystemseng.2016.08.024

[B16] Fan X. Kawamura K. Guo W. Xuan T. D. Lim J. Yuba N. (2018). A simple visible and near-infrared (V-NIR) camera system for monitoring the leaf area index and growth stage of Italian ryegrass. *Comput. Electron. Agric.* 144 314–323. 10.1016/j.compag.2017.11.025

[B17] Ferentinos K. P. (2018). Deep learning models for plant disease detection and diagnosis. *Comput. Electron. Agric.* 145 311–318. 10.1016/j.compag.2018.01.009

[B18] Ferreira A. d. S Freitas D. M. da Silva G. G. Pistori H. Folhes M. T. (2017). Weed detection in soybean crops using ConvNets. Comput. Electron. *Agriculture* 143 314–324. 10.1016/j.compag.2017.10.027

[B19] Grahn C. M. Benedict C. Thornton T. Miles C. (2015). Production of baby-leaf salad greens in the spring and fall seasons of Northwest Washington. *HortScience* 50 1467–1471. 10.21273/hortsci.50.10.1467 35581909

[B20] Greener J. G. Kandathil S. M. Moffat L. Jones D. T. (2022). A guide to machine learning for biologists. *Nat. Rev. Mol. Cell Bio.* 23 40–55. 10.1038/s41580-021-00407-0 34518686

[B21] Grinblat G. L. Uzal L. C. Larese M. G. Granitto P. M. (2016). Deep learning for plant identification using vein morphological patterns. *Comput. Electron. Agric.* 127 418–424. 10.1016/j.compag.2016.07.003

[B22] He K. Zhang X. Ren S. Sun J. (2016). “Deep residual learning for image recognition,” in *Proceedings of the IEEE Conference on Computer Vision and Pattern Recognition*, (Las Vegas). 10.1109/CVPR.2016.90

[B23] Hu Y. Wang L. Xiang L. Wu Q. Jiang H. (2018). Automatic non-destructive growth measurement of leafy vegetables based on kinect. *Sensors* 18:806. 10.3390/s18030806 29518958 PMC5876734

[B24] Huang G. Liu Z. van der Maaten L. Weinberger K. Q. Ieee. (2017). “Densely connected convolutional networks,” in *Proceedings of the IEEE Conference on Computer Vision and Pattern Recognition*, (Honolulu). 10.1109/CVPR.2017.243

[B25] Jiang Y. Li C. Paterson A. H. (2016). High throughput phenotyping of cotton plant height using depth images under field conditions. *Comput. Electron. Agric.* 130 57–68. 10.1016/j.compag.2016.09.017

[B26] Lati R. N. Filin S. Eizenberg H. (2013). Estimation of plants’ growth parameters via image-based reconstruction of their three-dimensional shape. *Agr. J.* 105 191–198. 10.2134/agronj2012.0305

[B27] Lauguico S. Concepcion R. Tobias R. R. Alejandrino J. Guia J. D. Guillermo M. (2020). “Machine vision-based prediction of lettuce phytomorphological descriptors using deep learning networks,” in *Proceedings of the IEEE International Conference on Humanoid, Nanotechnology, Information Technology, Communication and Control, Environment, and Management*, (Manila). 10.1109/HNICEM51456.2020.9400103

[B28] Liu F. Li G. Yang S. Yan W. He G. Lin L. (2020). Detection of heterogeneity on multi-spectral transmission image based on multiple types of pseudo-color maps. *Infrar. Phys. Techn.* 106:103285. 10.1016/j.infrared.2020.103285

[B29] Luo H. Cheng F. Yu H. Yi Y. (2021). SDTR: Soft decision tree regressor for tabular data. *IEEE Access* 9 55999–56011. 10.1109/access.2021.3070575

[B30] Ma J. Du K. Zhang L. Zheng F. Chu J. Sun Z. (2017). A segmentation method for greenhouse vegetable foliar disease spots images using color information and region growing. *Comput. Electron. Agric.* 142 110–117. 10.1016/j.compag.2017.08.023

[B31] Ma J. Li Y. Chen Y. Du K. Zheng F. Zhang L. (2019). Estimating above ground biomass of winter wheat at early growth stages using digital images and deep convolutional neural network. *Eur. J. Agr.* 103 117–129. 10.1016/j.eja.2018.12.004

[B32] Mohanty S. P. Hughes D. P. Salathé M. (2016). Using deep learning for image-based plant disease detection. *Front. Plant Sci.* 7:1419. 10.3389/fpls.2016.01419 27713752 PMC5032846

[B33] Mortensen A. K. Bender A. Whelan B. Barbour M. M. Sukkarieh S. Karstoft H. (2018). Segmentation of lettuce in coloured 3D point clouds for fresh weight estimation. *Comput. Electron. Agric.* 154 373–381. 10.1016/j.compag.2018.09.010

[B34] Nachtigall L. G. Araujo R. M. Nachtigall G. R. (2016). “Classification of apple tree disorders using convolutional neural networks,” in *Proceedings of the IEEE International Conference on Tools with Artificial Intelligence*, (San Jose, CA: IEEE). 10.1109/ICTAI.2016.0078

[B35] Sakamoto T. Gitelson A. A. Wardlow B. D. Arkebauer T. J. Verma S. B. Suyker A. E. (2012). Application of day and night digital photographs for estimating maize biophysical characteristics. *Prec. Agric.* 13 285–301. 10.1007/s11119-011-9246-1

[B36] Simonyan K. Zisserman A. (2014). Very deep convolutional networks for large-scale image recognition. *arXiv* [preprint]. 10.48550/arXiv.1409.1556 35895330

[B37] Song Y. S. Liang J. Y. Lu J. Zhao X. W. (2017). An efficient instance selection algorithm for k nearest neighbor regression. *Neurocomputing* 251 26–34. 10.1016/j.neucom.2017.04.018

[B38] Tackenberg O. (2007). A new method for non-destructive measurement of biomass, growth rates, vertical biomass distribution and dry matter content based on digital image analysis. *Ann. Bot.* 99 777–783. 10.1093/aob/mcm009 17353204 PMC2802942

[B39] Teobaldelli M. Rouphael Y. Fascella G. Cristofori V. Rivera C. M. Basile B. (2019). Developing an accurate and fast non-destructive single leaf area model for loquat (Eriobotrya japonica Lindl) Cultivars. *Plants* 8:230. 10.3390/plants8070230 31319530 PMC6681347

[B40] Tudela J. A. Hernández N. Pérez-Vicente A. Gil M. I. (2017). Growing season climates affect quality of fresh-cut lettuce. *Postharv. Bio. Techn.* 123 60–68. 10.1016/j.postharvbio.2016.08.013

[B41] Uzal L. C. Grinblat G. L. Namias R. Larese M. G. Bianchi J. S. Morandi E. N. (2018). Seed-per-pod estimation for plant breeding using deep learning. *Comput. Electron. Agric.* 150 196–204. 10.1016/j.compag.2018.04.024

[B42] Xiong J. Yu D. Liu S. Shu L. Wang X. Liu Z. (2021). A review of plant phenotypic image recognition technology based on deep learning. *Electronics* 10:1. 10.3390/electronics10010081

[B43] Xiong X. Yu L. Yang W. Liu M. Jiang N. Wu D. (2017). A high-throughput stereo-imaging system for quantifying rape leaf traits during the seedling stage. *Plant Methods* 13:7. 10.1186/s13007-017-0157-7 28163771 PMC5282657

[B44] Xu C. Mou B. (2015). Evaluation of lettuce genotypes for salinity tolerance. *HortScience* 50 1441–1446. 10.21273/hortsci.50.10.1441 35581909

[B45] Yoosefzadeh N. M. (2021). *Using advanced proximal sensing and genotyping tools combined with bigdata analysis methods to improve soybean yield. [master’s thesis].* Canada: University of Guelph.

[B46] Zhang L. Verma B. Stockwell D. Chowdhury S. (2018). Density weighted connectivity of grass pixels in image frames for biomass estimation. *Exp. Syst. Appl.* 101 213–227. 10.1016/j.eswa.2018.01.055

[B47] Zhang L. Xu Z. Xu D. Ma J. Chen Y. Fu Z. (2020). Growth monitoring of greenhouse lettuce based on a convolutional neural network. *Hortic. Res.* 7:124. 10.1038/s41438-020-00345-6 32821407 PMC7395764

